# Особенности обоняния и размеры обонятельных луковиц при синдроме Кальмана

**DOI:** 10.14341/probl13216

**Published:** 2023-05-11

**Authors:** К. Д. Кокорева, И. С. Чугунов, В. П. Владимирова, Т. Е. Иванникова, В. П. Богданов, О. Б. Безлепкина

**Affiliations:** Национальный медицинский исследовательский центр эндокринологии; Национальный медицинский исследовательский центр эндокринологии; Национальный медицинский исследовательский центр эндокринологии; Национальный медицинский исследовательский центр эндокринологии; Национальный медицинский исследовательский центр эндокринологии; Национальный медицинский исследовательский центр эндокринологии

**Keywords:** синдром Кальмана, аносмия, гипосмия, ольфактометрическое исследование, гипоплазия обонятельных луковиц, аплазия обонятельных луковиц

## Abstract

**ОБОСНОВАНИЕ:**

ОБОСНОВАНИЕ. В подавляющем большинстве пациенты с синдромом Кальмана отмечают неспособность различать запахи, что помогает своевременно установить диагноз. Некоторые из пациентов с синдромом Кальмана не предъявляют подобных жалоб, но при помощи ольфактометрии с использованием специальных наборов пахучих веществ у них выявляются нарушения обоняния. Нарушения обоняния часто отмечаются у пациентов с гипоплазией или аплазией одной или обеих обонятельных луковиц (ОЛ). Характер взаимосвязи размеров обонятельных луковиц и гипоили аносмии по результатам ольфактометрии у пациентов с ВИГГ в настоящее время мало изучен.

**ЦЕЛЬ:**

ЦЕЛЬ. Изучить размеры ОЛ и обонятельную функцию у детей с ВИГГ. Установить наличие взаимосвязи между размерами ОЛ и обонятельной функцией.

**МАТЕРИАЛЫ И МЕТОДЫ:**

МАТЕРИАЛЫ И МЕТОДЫ. Одноцентровое одномоментное сравнительное исследование. В исследование включены 34 пациента. Основную группу составили 19 детей с гипогонадотропным гипогонадизмом (15 — с синдромом Кальмана, 4 — с нормосмическим гипогонадотропным гипогонадизмом). Всем пациентам проводилась МРТ головного мозга с оценкой размеров ОЛ, ольфактометрический тест (Sniffin’ Sticks Test) и молекулярно-генетические исследования. Контрольную группу составили 15 детей, у которых при проведении МРТ орбит дополнительно оценивали размеры ОЛ.

**РЕЗУЛЬТАТЫ:**

РЕЗУЛЬТАТЫ. Из 19 пациентов с ВИГГ нормальные размеры ОЛ имелись только у 1 пациента. У детей с гипогонадизмом высота и ширина ОЛ оказались достоверно меньше (p<0,01) в сравнении с контролем. Медиана высоты правой луковицы (ПЛ) у пациентов с ВИГГ составила 1,0 мм [0,2; 1,8] против 3,0 [2,5; 3,2] в контрольной группе, медиана ширины ПЛ — 1,0 мм [0,2; 1,9] против 2,5 [2,0; 3,0], медиана высоты левой луковицы (ЛЛ) у пациентов с ВИГГ — 0,8 мм [0,0; 1,2] против 3,0 [2,7; 3,2], медиана ширины ЛЛ — 0,8 мм [0,0; 1,2] против 2,5 [2,0; 3,0]. Выявлена корреляция между высотой (r=0,59) и шириной (r=0,67) левой ОЛ и результатами ольфактометрического теста (p<0,05). У 4 пациентов субъективная оценка обонятельной функции не совпала с данными ольфактометрического исследования.

**ЗАКЛЮЧЕНИЕ:**

ЗАКЛЮЧЕНИЕ. Ольфактометрия позволила выявить нарушения обоняния у 78,5% пациентов с изолированным гипогонадотропным гипогонадизмом (15 из 19 пациентов), при этом субъективно на нарушения обоняния жаловались только 11 из 19 пациентов. Впервые в РФ представлены размеры ОЛ у пациентов с изолированным гипогонадотропным гипогонадизмом. У 94,7% пациентов вне зависимости от нарушения обонятельной функции отмечалась гипоплазия и/или аплазия одной или обеих ОЛ. Чаще всего встречалась гипоплазия обеих луковиц (36,8%), изменения ольфакторных луковиц (гипоплазия или аплазия) с одной стороны имелись у 31,6% пациентов.

## Abstract (en)

**BACKGROUND:**

BACKGROUND: The majority of Kallmann patients have anosmia or hyposmia. This is how the disease is diagnosed. Some of them don’t have such complaints but olfactory dysfunction is diagnosed via olfactometry. Nowadays there is the lack of information about correlation between olfactometry results and subjective complaints. Correlation between olfactory bulbs size and olfactory dysfunction has been little studied.

**AIM:**

AIM: To explore olfactory bulb size and olfactory function in patients with congenital isolated hypogonadotropic hypogonadism. To correlate olfactory bulb sizes and smell test scores.

**MATERIALS AND METHODS:**

MATERIALS AND METHODS: Single-centre comparative study. 34 patients were included. The main group consisted of 19 patients with hypogonadotropic (15 –with Kallmann syndrome, 4 — with normosmic hypogonadism). Olfactory bulbs MRI were provided to all the patients, olfactory test (Sniffin’ Sticks Test) and molecular-genetic studies were provided in all patients with hypogonadism. Control group consisted of 15 patients who were provided with orbits MRI. Olfactory bulbs were evaluated additionally in them.

**RESULTS:**

RESULTS: Normal size of olfactory bulbs were only in 1 patient with hypogonadism. Olfactory bulbs height and width were significantly smaller in patients with hypogonadism in comparison with control group (p<0.01). Height median of right bulb was 1.0 mm [0.2; 1.8] in patients from the main group vs. 3.0 [2.5; 3.2] in controls, width median of right bulb was 1.0 mm [0.2; 1.9] in patients from the main group vs. 2.5 [2.0; 3.0] in controls. Height median of left bulb was 0.8 mm [0.0; 1.2] in patients from the main group vs. 3.0 [2.7; 3.2] in controls, width median of left bulb was 0.8 mm [0.0; 1.2] in patients from the main group vs. 2.5 [2.0; 3.0] in controls. Correlation has been established between left bulb height (r=0.59) and width (r=0.67) and olfactometry results (p<0.05). 4 patients had no anosmia complaints but had olfactory dysfunction according to Sniffin’ Sticks Tests.

**CONCLUSION:**

CONCLUSION: Olfactometry was able to diagnose olfactory dysfunction in 78.5% (i.e. in 15 out of 19 patients with congenital isolated hypogonadotropic hypogonadism. However, anosmia complaints had only 11 out of 19 patients. It is the first results of olfactory bulb sizes in patients with hypogonadotropic hypogonadism in Russia. Uni — or bilateral hypoor aplasia were diagnosed in 94.7% patients with hypogonadism regardless of olfactory dysfunction. Bilateral olfactory bulbs hypoplasia were the most common MRI-finding (36.8%). Unilateral hypoor aplasia was diagnosed in 31.6% patients.

## ОБОСНОВАНИЕ

Изолированный гипогонадотропный гипогонадизм — редкая врожденная патология. В половине случаев, а по некоторым данным до 2/3 случаев врожденного изолированного гипогонадотропного гипогонадизма (ВИГГ) [1], проявляются синдромом Кальмана. Впервые данный синдром был описан в 1944 г. Среди мальчиков частота синдрома составляет 1 на 4000–8000 [1][2], а среди девочек — 1 на 40 000 [2]. Вариантные замены в более чем 40 генах ассоциированы с развитием гипогонадизма. Различные клинические проявления синдрома в ряде случаев обусловлены генетическим полиморфизмом заболевания: так, например, при сочетании гипогонадизма с аномалиями развития конечностей приоритетным геном для поиска вариантных замен является ген фактора роста фибробластов 1-го типа (FGFR1), а при бимануальной синкинезии и аносмии — ген белка аносмина (KAL1) [3]. Частыми клиническими проявлениями ВИГГ среди мальчиков являются крипторхизм и/или микропения. Считается, что они сопутствуют наиболее тяжелым формам гипогонадизма [3].

Пациенты с синдромом Кальмана, как правило, неспособны различать «тонкие» запахи, но могут слышать такие резкие запахи, как запах ацетона, аммиака или мяты, что, по-видимому, связано со способностью этих запахов раздражать рецепторы тройничного нерва [4]. Золотым стандартом оценки обонятельной функции является метод хемосенсорных вызванных потенциалов, который часто применяют в оториноларингологии и неврологии [4–6]. При воздействии на ольфакторные рецепторы у пациентов с нарушением обоняния определяют меньшую амплитуду вызванных потенциалов [3, 4]. Однако в связи с технической сложностью выполнения метода в рутинной практике он, как правило, не проводится [6]. Более доступными, наиболее часто применяемыми и стандартизованными методами оценки обоняния являются следующие психофизические методы: Пенсилванский тест (University of Pennsylvania Smell Identification Test, UPSIT, USA) и Sniffin' Sticks Test (Germany). Оба теста помогают определить степень нарушения ольфакторной функции, а также оценить ее улучшение или ухудшение с течением времени, если это необходимо [6]. В нашем исследовании применялась укороченная версия Sniffin’ Sticks Test, которая предполагает определение 12 стандартизированных запахов, при ответе пациент выбирает один из 4 продолженных вариантов. Такой метод оценки обоняния называют методом принудительного выбора. Sniffin’ Sticks Test неоднократно использовался для оценки обонятельной функции у взрослых пациентов и подростков с синдромом Кальмана [7][8].

Известно, что при проведении магнитно-резонансной томографии (МРТ) у пациентов с синдромом Кальмана отмечается отсутствие обонятельных луковиц (ОЛ) (аплазия) или уменьшение их размеров (гипоплазия) [7]. Такие изменения могут носить двусторонний характер или затрагивать только одну ОЛ. ОЛ располагаются на нижней поверхности лобных долей в передней черепной ямке. Они являются уникальным органом, функция которых зависит от размера, что предполагает наличие взаимосвязи между размерами луковиц и нарушением обоняния. Однако на настоящий момент объективных данных о размерах ОЛ у детей с синдромом Кальмана в отечественной литературе не представлено, а наличие корреляционной связи между размерами ОЛ и нарушением обонятельной функции не всегда подтверждается [9].

## ЦЕЛЬ ИССЛЕДОВАНИЯ

Изучить размеры ОЛ и обонятельную функцию у детей с ВИГГ. Установить наличие взаимосвязи между размерами ОЛ и обонятельной функцией.

## МАТЕРИАЛЫ И МЕТОДЫ

Место и время проведения исследования

Место проведения. Исследование проведено на базе ФГБУ «НМИЦ эндокринологии» Минздрава России.

Время исследования. Исследование проводилось в течение 2 лет, с ноября 2020 г. по ноябрь 2022 г.

Изучаемые популяции (одна или несколько)

Популяции. В исследование включены 34 ребенка: 19 детей (16 мальчиков и 3 девочки) с гипогонадотропным гипогонадизмом (основная группа) и 15 детей (9 мальчиков и 6 девочек) — контрольная группа. Группы были сопоставимы по полу, возрасту, показателям роста и массы тела (p>0,05), но не по стадии полового развития (p<0,05).

Критерии включения в основную группу: мальчики старше 14 лет, девочки старше 13 лет с диагнозом «гипогонадотропный гипогонадизм», подписание родителями информированного согласия на участие в исследовании.

Критерии невключения: множественный дефицит гормонов гипофиза, наличие заболеваний, которые могут сопровождаться аносмией (нейродегенеративные заболевания, голопрозэнцефалия, аллергический ринит, оперативные вмешательства в области носа, травмы головы в анамнезе), умственная отсталость, отказ от участия в исследовании.

Критерии включения в группу контроля: мальчики старше 14 лет, девочки старше 13 с эндокринной офтальмопатией, которым проводилось МРТ орбит с дополнительной оценкой размеров ОЛ, подписание родителями информированного согласия.

Способ формирования выборки из изучаемой популяции (или нескольких выборок из нескольких изучаемых популяций)

Сплошной способ формирования выборки.

Дизайн исследования

Одноцентровое одномоментное сравнительное исследование.

Методы

Всем пациентам проведена МРТ головы с оценкой размеров ОЛ: исследование проводилось на аппарате «Magnetom Harmony» (Siemens, Германия) c напряженностью магнитного поля 1,5 Тесла в Т1- и Т2-взвешенных режимах. Средний размер высоты ОЛ у здорового человека составляет 2,8±0,3 мм, а ширины — 4,7±0,5 мм [10]. Снижение обоих параметров (и высоты, и ширины) луковиц более чем на 50%, расценивалось как гипоплазия. Отсутствие луковиц в ольфакторной ямке расценивалось как аплазия.

Ольфактометрическое исследование проводилось методом принудительного выбора посредством Sniffin’ Sticks Screening 12 Item test (Burghart Messtechnik, Германия). Тестирование предполагало определение пациентом 12 различных запахов и соотнесение их с предложенными. Sniffin’ Sticks Test представляет собой набор из 12 контейнеров в форме карандаша, наконечник каждого из которых пропитан жидкостью со специфическим запахом. Пациенты были ознакомлены с правилами проведения теста [11]. Перед проведением теста помещение тщательно проветривалось. После того, как пациент закрыл глаза, исследователь подносил контейнеры на расстояние 2 см от носа пациента и удерживал в течение 2–3 с. Выдерживались предписанные паузы между исследованием разных запахов. Обозначения некоторых запахов были заменены на более легко узнаваемые российскими детьми: например, запах лакрицы был заменен на «сироп от кашля» [12]. В зависимости от количества набранных баллов устанавливалась нормосмия (11–12 баллов), гипосмия (9–10 баллов) или аносмия (8 баллов и ниже).

Молекулярно-генетическое исследование проведено всем пациентам с гипогонадизмом в ФГБУ «НМИЦ эндокринологии» Минздрава России. 17 пациентам исследование проводилось методом секвенирования следующего поколения (NGS) с применением авторской панели «Гипогонадотропный гипогонадизм» (технология Ion Ampliseq™ Custom DNA Panel, Thermo Scientific, Waltham, MA, USA), содержащий праймеры для мультиплексной полимеразной цепной реакции и секвенирования кодирующих последовательностей следующих 53 генов: ANOS1, BBS1, BBS10, BBS12, BBS2, BBS4, BBS7, BBS9, CHD7, WDR11, DNMT3L, DUSP6, FEZF1, FGF17, FGF8, FGFR1, FLRT3, GNRH1, GNRHR, HS6ST1, IL17RD, INSL3, KISS1, KISS1R, LEP, LEPR, LHB, MC4R, MKKS, MKRN3, MKS1, MTTP, NR0B1, NSMF, NTRK2, PCSK1, PNPLA6, POLR3A, POLR3B, PROK2, PROKR2, PROP1, RBM28, RNF216, RXFP2, SEMA3A, SH2B1, SIM1, SOX10, SPRY4, TAC3, TACR3, TTC8. Интерпретация вариантов нуклеотидной последовательности проводилась согласно руководству по интерпретации данных последовательности ДНК человека, полученных методами массового параллельного секвенирования. Двум пациентам проводилось полногеномное секвенирование с неглубоким покрытием (средняя глубина покрытия – 96x) для выявления протяженных делеций (пациенты 4 и 12 с ВИГГ и ихтиозом). Номера пациентам с гипогонадизмом присваивались в порядке их включения в исследование.

Статистический анализ

Данные представлены в виде значения медианы и интерквартильного размаха: Me [Q1; Q3]. Для выявления статистически значимых различий между двумя независимыми группами был использован критерий Манна–Уитни. Корреляционный анализ проводился методом Спирмена. Уровень р<0,05 считался статистически значимым. Для нивелирования проблемы множественных сравнений применялся p-value, скорректированный поправкой Бонферрони. Расчет данных производился с помощью статистического пакета Statistica 12 (StatSoft inc., США).

Этическая экспертиза

Проведение исследования одобрено локальным Этическим комитетом ФГБУ «НМИЦ эндокринологии» Минздрава России. Протокол № 18 от 11/10/2020.

## РЕЗУЛЬТАТЫ

В группу детей с ВИГГ включены 19 детей (16 мальчиков и 3 девочки, медиана возраста 15,9 года [ 14,4; 16,3]). На нарушения обоняния предъявляли жалобы 11 детей, у которых проведение ольфактометрического теста подтвердило аносмию. Восемь детей не предъявляли жалоб на нарушения обоняния, однако при проведении ольфактометрического теста у 3 из них отмечалась аносмия, у 1 — гипосмия, у 4 — нормосмия. Таким образом, после проведения ольфактометрического исследования у 15 детей диагностирован синдром Кальмана. В таблице 1 представлена подробная характеристика пациентов с ВИГГ.

Молекулярно-генетические исследования выявили вариантные замены у 8 пациентов: у 2 — патогенные замены, у 1 — вероятно патогенный вариант, у остальных пациентов — замены с неизвестной клинической значимостью. Наиболее часто вариантные замены определялись в гене FGFR1 (у 4 из 8 пациентов), у пациента 5 вариантная замена сопровождалась развитием микропении, у пациента 6 — крипторхизмом, а у пациента 15 — сочетанием крипторхизма с микропенией. Характерных фенотипических проявлений (расщелина губы и неба, аномалии развития нижних конечностей) при вариантных заменах в гене FGFR1 не наблюдалось.

У двух мальчиков выявлены вариантные замены в генах CHD7 (пациент 8) и MKRN3 (пациент 1). У пациента 3 с бимануальной синкинезией имелись две вариантные замены в гене KAL1. У пациентки 14 выявлена вариантная замена в гене FGF17. У пациентов 4 и 12 имел место ихтиоз, что позволило заподозрить у них микроделеционный синдром. Диагностика микроделеционного синдрома требует проведения хромосомного микроматричного анализа [13] или полногеномного секвенирования с неглубоким покрытием. У обоих пациентов полногеномное секвенирование выявило патогенные протяженные делеции в гемизиготном состоянии участка Х хромосомы: у пациента 4 — с приблизительными границами chrX:6637902-8623178 размером 1985276 нуклеотидных последовательностей и генами PUDP, STS, VCX, PNPLA4, VCX2, VCX3B, ANOS1, а у пациента 12 — делеция с приблизительными границами chrX:5892507-9033380 и размером 3140873 нуклеотидных последовательностей, включающая гены ANOS1, FAM9A, FAM9B, NLGN4X, PNPLA4, PUDP, STS, VCX, VCX2, VCX3A, VCX3B.

Проведенное МРТ-исследование ОЛ (n=34) выявило достоверное снижение их размеров у пациентов с гипогонадизмом по сравнению с группой контроля (табл. 2).

По результатам МРТ у 18 из 19 пациентов с ВИГГ наблюдалось снижение размеров ОЛ или их полное отсутствие. Практически у каждого третьего пациента отмечалась гипоплазия обеих луковиц (7 пациентов). Реже отмечалась односторонняя гипоплазия (27,8%), двусторонняя аплазия (16,6%), гипоплазия одной луковицы в сочетании с аплазией другой (11,1%). Аплазия одной луковицы имелась у 1 пациента. На рисунках 1 и 2 выделена область обонятельных луковиц нормального размера у пациентки из контрольной группы и область с аплазированными ОЛ у пациента с синдромом Кальмана.

Проведен углубленный сравнительный анализ размеров ОЛ у пациентов с синдромом Кальмана и пациентов с гипогонадизмом без нарушения обоняния и выявлена достоверная разница в высоте ОЛ (табл. 3).

Проведенный корреляционный анализ у пациентов с ВИГГ выявил взаимосвязь между результатами ольфактометрии и размерами левой луковицы (для высоты r=0,59, для ширины r=0,67, p<0,05). Для правой луковицы подобной взаимосвязи установлено не было.

Интерес вызывают четыре пациента, не предъявлявшие первоначально жалоб на нарушения обоняния. Проведение ольфактометрического теста выявило у них различную степень снижения обоняния (от 2 до 8 баллов). У всех 4 пациентов отмечались изменения ОЛ по данным МРТ. У двух из них (пациенты 15 и 17) обнаружены вариантные замены в гене FGFR1. Вероятно патогенная замена в этом гене, приводящая к терминации экспрессии гена и образованию белка с меньшей молекулярной массой, у пациента 15 была ассоциирована с односторонним крипторхизмом, микропенией и аплазией обеих ОЛ. У пациентки 17 с вариантной заменой неизвестной клинической значимости в гене FGFR1 не отмечалось особенностей фенотипа, по результатам ольфактометрии она набрала 8 баллов из 12 (аносмия), а по данным МРТ выявлена гипоплазия обеих ОЛ. У двух оставшихся пациентов с аносмией не обнаружено вариантных замен, по данным МРТ у обоих отмечалась односторонняя гипоплазия ОЛ, которая в одном случае сопровождалась гипоспадией и тугоухостью, а в другом — двусторонним крипторхизмом.

**Table:** Таблица 1. Характеристика пациентов с врожденным изолированным гипогонадотропным гипогонадизмом Примечание: СК — синдром Кальмана; НВИГГ — нормосмический врожденный изолированный гипогонадотропный гипогонадизм; ВЗ — вариантная замена, П — патогенный; ВП — вероятно патогенный; НКЗ — неизвестной клинической значимости.

Пациент	Возраст, лет	Пол	Жалобы на нарушения обоняния	Ольфактометрия	Диагноз	МРТлуковиц	Особенностифенотипа	Молекулярно-генетическое исследование
Результат	Баллы	ВЗ	Аминокислотная замена	Патогенность ВЗ
1	16,4	М	Нет	Норма	12	НВИГГ	Гипоплазия обеих ОЛ	Нет	MKRN3(NM 005664)с.1315A>G	p.Ser439Gly	НКЗ
2	14,3	Ж	Да	Аносмия	1	СК	Гипоплазия обеих ОЛ	Нет	Не выявлено
3	15,5	М	Да	Аносмия	2	СК	Аплазия ПЛ,гипоплазия ЛЛ	Бимануальнаясинкинезия, двусторонний крипторхизм	KAL1(NM00021.6)	c.708C>A	p.His236Gln	НКЗ
c710_715del	p.W237_T239delinsS	НКЗ
4	14,3	М	Да	Аносмия	1	СК	Гипоплазия ПЛ, аплазия ЛЛ	Ихтиоз	Делеция chrX:6637902-8623178	П
5	16,3	М	Да	Аносмия	2	СК	Гипоплазия обеих ОЛ	Микропения	FGFR1(NM 023110.3)c.709G>A	p.Gly237Ser	П
6	15,5	М	Нет	Норма	11	НВИГГ	Гипоплазия ЛЛ	Одностороннийкрипторхизм	FGFR1(NM 023110.3) с.1711del	p.Glu571SerfsTer61	П
7	17,5	М	Да	Аносмия	2	СК	Гипоплазия обеих ОЛ	Нет	Не выявлено
8	17,2	М	Да	Аносмия	2	СК	Гипоплазия обеих ОЛ	Нет	CHD7 (NM 017780.4)5′-UTR, с.15G>A	НКЗ
9	14,3	М	Нет	Аносмия	6	СК	Гипоплазия ПЛ	Двустороннийкрипторхизм	Не выявлено
10	14,2	М	Да	Аносмия	4	СК	Гипоплазия ПЛ	Одностороннийкрипторхизм	Не выявлено
11	16,1	М	Нет	Аносмия	2	СК	Гипоплазия ЛЛ	Гипоспадия,тугоухость	Не выявлено
12	14,9	М	Да	Аносмия	2	СК	Аплазия обеих ОЛ	Ихтиоз, двусторонний крипторхизм,микропения, ожирение, нарушение поведения	Делеция chrX:5892507-9033380	П
13	16,5	М	Да	Аносмия	2	СК	Гипоплазия ЛЛ	Нет	Не выявлено
14	14,5	Ж	Нет	Норма	12	НВИГГ	Гипоплазия обеих ОЛ	Нет	FGF17(NM 003867.4)c.359C>T	p.Pro120Leu	НКЗ
15	15,9	М	Нет	Аносмия	3	СК	Аплазия обеих ОЛ	Односторонний крипторхизм, микропения	FGFR1(NM 023110.3)c.1997G>А	p.Trp666Ter	ВП
16	14,1	М	Да	Аносмия	1	СК	Аплазия обеих ОЛ	Микропения	Не выявлено
17	16,1	Ж	Нет	Аносмия	8	СК	Гипоплазия обеих ОЛ	Нет	FGFR1(NM 023110.3)c.2292+16C>T		НКЗ
18	16,1	М	Да	Аносмия	0	СК	Аплазия ЛЛ	Нет	Не выявлено
19	16,3	М	Нет	Норма	11	НВИГГ	Нормальный объем ОЛ	Нет	Не выявлено

**Table:** Таблица 2. Размеры обонятельных луковиц по данным МРТ Скорректированный поправкой Бонферрони p-value = 0,0125

Параметр Me [Q1; Q3], мм	Основная группа (n=19)	Контрольная группа (n=15)	p
Правая луковица	Высота	1,0 [ 0,2; 1,8]	3,0 [ 2,5; 3,2]	<0,01
Ширина	1,0 [ 0,2; 1,9]	2,5 [ 2,0; 3,0]	<0,01
Левая луковица	Высота	0,8 [ 0,0; 1,2]	3,0 [ 2,7; 3,2]	<0,01
Ширина	0,8 [ 0,0; 1,2]	2,5 [ 2,0; 3,0]	<0,01

**Table:** Таблица 3. Размеры обонятельных луковиц у детей с синдромом Кальмана и у детей с НВИГГ Скорректированный поправкой Бонферрони p-value = 0,0125

Параметр Me [Q1; Q3], мм	Синдром Кальмана (n=15)	НВИГГ (n=4)	p
Правая луковица	Высота	0,9 [ 0,0; 1,2]	1,5 [ 0,8; 2,5]	<0,01
Ширина	1,0 [ 0,0; 1,7]	1,5 [ 0,9; 2,2]	>0,05
Левая луковица	Высота	0,8 [ 0,0; 1,2]	1,0 [ 0,8; 1,7]	<0,01
Ширина	0,3 [ 0,0; 1,2]	1,0 [ 0,6; 2,3]	>0,05

**Figure:**
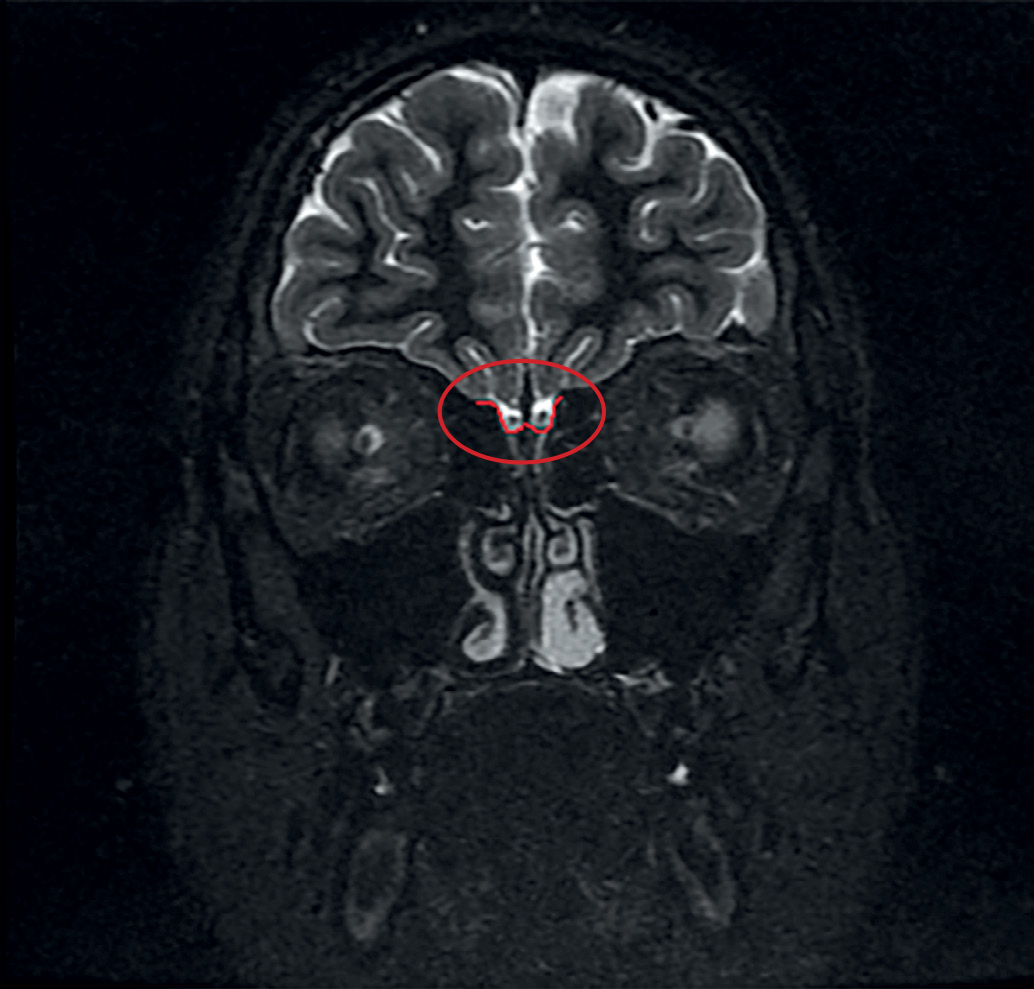
Рисунок 1. Нормальные размеры обонятельных луковиц у девочки контрольной группы.

**Figure:**
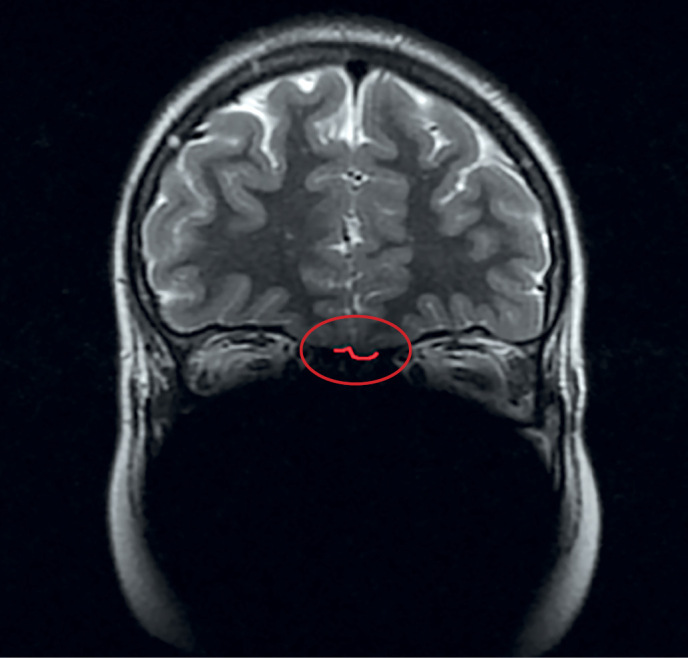
Рисунок 2. Аплазия обонятельных луковиц у пациента с синдромом Кальмана.

## ОБСУЖДЕНИЕ

Сочетание гипогонадотропного гипогонадизма с нарушением обоняния при синдроме Кальмана объясняется нарушением внутриутробной миграции ольфакторных нейронов и нейронов, секретирующих гонадотропин-рилизинг-гормон (ГнРГ). Ольфакторные и ГнРГ-секретирующие нейроны совместно закладываются в ольфакторной плакоде — инвагинации эктодермы, которая располагается вне головного мозга и впоследствии формирует назальный эпителий. Аксоны обонятельных нейронов проникают через решетчатую пластинку в передний мозг. Вдоль аксонов мигрируют ГнРГ-секретирующие нейроны. Контакт окончаний аксонов с клетками переднего мозга приводит к прекращению митотических делений клеток, их дифференцировке в нейробласты и образованию ОЛ. Данный процесс дифференцировки невозможен без аносмина и фактора роста фибробластов 1-го типа соответственно, патогенные вариантные замены в генах, кодирующих белок аносмин KAL1 (он же ANOS1) и фактор роста фибробластов 1-го типа FGFR1, могут быть ассоциированы с недоразвитием ОЛ [14].

У пациента 12 с гипогонадизмом, ихтиозом, микропенией, ожирением и нарушением поведения был диагностирован синдром потери генных последовательностей, который связан с выпадением участка короткого плеча Х-хромосомы. По результатам полногеномного секвенирования с неглубоким покрытием выявлена делеция участка Х-хромосомы, содержащего гены ANOS1, STS, NLGN4X и др. Выпадение гена ANOS1 (он же KAL1) ассоциировано с развитием Х-сцепленного гипогонадотропного гипогонадизма [15] и аносмии, выпадение гена стероидной сульфатазы STS — с ихтиозом [16], а гена NLGN4X — c аутизмом [17], что, вероятно, объясняет нарушения поведения. У пациента 4 была диагностирована меньшая по протяженности делеция Х хромосомы с генами ANOS1, STS, VCX, PNPLA4, VCX2, VCX3B, PUDP, чем объясняется наличие у пациента ихтиоза с гипогонадизмом без нарушений поведения.

Синдром Кальмана — наиболее частая [18], но не единственная возможная причина нарушения закладки ОЛ: недоразвитие ОЛ наблюдается при сосудистых аномалиях, голопрозэнцефалии, септооптической дисплазии, врожденной изолированной агенезии ОЛ и др. [19]. Таким образом, изменение размеров ОЛ может являться одним из признаков гипогонадизма после исключения других причин гипоплазии ОЛ.

По результатам исследований у пациентов с синдромом Кальмана чаще выявлялась полная аплазия обеих луковиц: так, в исследовании Т. Hacquart et al. [20] данные структуры головного мозга отсутствовали у 14 из 19 пациентов с синдромом Кальмана, а в исследовании Yu et al. аплазия отмечалась у 27 из 28 пациентов [21]. В нашем исследовании двусторонняя аплазия отмечалась только у 3 пациентов. В целом, как среди пациентов с синдромом Кальмана, так и среди пациентов с гипогонадизмом без нарушения обоняния, чаще отмечалась гипоплазия обеих луковиц (у 5 из 15 детей с синдромом Кальмана и у 2 из 4 детей с нормосмическим гипогонадизмом).

В нашем исследовании было 4 пациента с нормосмическим гипогонадизмом, никто из них не предъявлял жалоб на гипо- или аносмию, у всех по результатам ольфактометрического теста была подтверждена нормосмия. У 2 из них отмечалась гипоплазия обеих луковиц, у 1 — односторонняя гипоплазия. Эти результаты согласуются с данными зарубежных исследований: по результатам исследования В. Yu et al., проведенного в 2022 г., изменения ольфакторного аппарата у пациентов с гипогонадизмом без нарушения обоняния определялись у 9 из 36 пациентов (25%) [21]. Вероятнее всего, ненарушенная обонятельная функция у пациентов с гипоплазией одной или обеих луковиц объясняется сохранным остаточным объемом другой луковицы или обеих структур. Известно, что обонятельная функция у нормосмических пациентов с изменениями ольфакторного аппарата со временем может ухудшиться [21].

В большинстве работ установлена корреляция между объемом ОЛ и результатами Sniffin’ Sticks Test [7][8]. По результатам нашей работы установлена взаимосвязь между результатами теста и размерами левой ОЛ. Причина отсутствия взаимосвязи между обонятельной функцией и размерами правой луковицы на настоящий момент неизвестна и требует проведения дальнейших исследований.

## ЗАКЛЮЧЕНИЕ

Впервые в РФ представлены размеры ОЛ по данным МРТ-исследования у пациентов с ВИГГ. У 94,7% пациентов вне зависимости от нарушения обонятельной функции отмечались гипоплазия или аплазия ОЛ.

## ДОПОЛНИТЕЛЬНАЯ ИНФОРМАЦИЯ

Информация о конфликте интересов. Авторы декларируют отсутствие конфликта интересов.

Информация о финансировании. Работа проведена в рамках темы госзадания 123021000045-4 «Генетическая персонификация редких вариантов задержки роста и полового развития у детей».

Участие авторов. Кокорева К.Д., Иваникова Т.Е., Чугунов И.С — поисково-аналитическая работа и подготовка финальной версии статьи; Богданов В.П. — проведение молекулярно-генетического исследования; Владимирова В.П. — проведение магнитно-резонансной томографии пациентам из контрольной и основной групп; Безлепкина О.Б. — редактирование текста, внесение ценных замечаний. Все авторы одобрили финальную версию статьи перед публикацией, выразили согласие нести ответственность за все аспекты работы, подразумевающую надлежащее изучение и решение вопросов, связанных с точностью или добросовестностью любой части работы.
